# Oral phosphatidylcholine pretreatment alleviates the signs of experimental rheumatoid arthritis

**DOI:** 10.1186/ar2651

**Published:** 2009-03-18

**Authors:** Gabor Erős, Saleh Ibrahim, Nikolai Siebert, Mihály Boros, Brigitte Vollmar

**Affiliations:** 1 Institute for Experimental Surgery, University of Rostock, Schillingallee 69a, Rostock D-18057, Germany; 2 Institute of Surgical Research, University of Szeged, Pécsi u. 6, Szeged H-6720, Hungary; 3 Immunogenetics Group, University of Rostock, Schillingallee 70, Rostock D-18057, Germany

## Abstract

**Introduction:**

Phosphatidylcholine and phosphatidylcholine-derived metabolites exhibit anti-inflammatory properties in various stress conditions. We hypothesized that dietary phosphatidylcholine may potentially function as an anti-inflammatory substance and may decrease inflammatory activation in a chronic murine model of rheumatoid arthritis (collagen-induced arthritis).

**Methods:**

The experiments were performed on male DBA1/J mice. In groups 1 to 3 (n = 10 each), collagen-induced arthritis was induced by administration of bovine collagen II. In group 2 the animals were fed *ad libitum *with phosphatidylcholine-enriched diet as a pretreatment, while the animals of group 3 received this nourishment as a therapy, after the onset of the disease. The severity of the disease and inflammation-linked hyperalgesia were evaluated with semiquantitative scoring systems, while the venular leukocyte–endothelial cell interactions and functional capillary density were assessed by means of *in vivo *fluorescence microscopy of the synovial tissue. Additionally, the mRNA expressions of cannabinoid receptors 1 and 2, TNFα and endothelial and inducible nitric oxide synthase were determined, and classical histological analysis was performed.

**Results:**

Phosphatidylcholine pretreatment reduced the collagen-induced arthritis-induced hypersensitivity, and decreased the number of leukocyte–endothelial cell interactions and the extent of functional capillary density as compared with those of group 1. It also ameliorated the tissue damage and decreased inducible nitric oxide synthase expression. The expressions of the cannabinoid receptors and TNFα were not influenced by the phosphatidylcholine intake. Phosphatidylcholine-enriched food administrated as therapy failed to evoke the aforementioned changes, apart from the reduction of the inducible nitric oxide synthase expression.

**Conclusions:**

Phosphatidylcholine-enriched food as pretreatment, but not as therapy, appears to exert beneficial effects on the morphological, functional and microcirculatory characteristics of chronic arthritis. We propose that oral phosphatidylcholine may be a preventive approach in ameliorating experimental rheumatoid arthritis-induced joint damage.

## Introduction

Rheumatoid arthritis (RA) reduces the health-related quality of life and imposes a substantial burden on both the individual and society [1]. The generalized chronic inflammation profoundly affects the function of several organ systems [2] and leads to symmetric, erosive skeletal changes, especially in the minor joints. Although the pathomechanism is still unclear, a number of data suggest that inflammatory mediators from the synovium play central roles in secondary structural bone damage [3,4]. By means of intravital microscopy (IVM), it has been shown that the granulocytes are the first major cell population recruited to the inflamed joints during the early phase of experimental RA [5]. The ensuing tissue destruction can be ascribed, at least partly, to leukocyte extravasation and the interference of activated synovial polymorphonuclear (PMN) granulocytes with other infiltrating immune cells and their products.

Many different disease-modifying antirheumatic drugs have been used to date, but the shaping of optimal therapy is difficult – mainly due to the prolonged application, side effects and costs of different agents [6,7]. In this respect, targeted nutritional interventions have many advantages, and various experimental and clinical data have indicated that dietary phosphatidylcholine (PC) may potentially function as an anti-inflammatory substance [8-11]. PC, a ubiquitous component of biological membranes, has additionally been demonstrated to increase the tissue tolerance in experimental models of ischemia and hypoxia [12-14]. The notion that PC may be anti-inflammatory is supported by the finding that PC metabolites with an alcoholic moiety in the molecule (choline, *N,N*-dimethylethanolamine and *N*-methylethanolamine) inhibit the reactive oxygen species-producing activity of isolated PMN granulocytes [15].

On the above basis, we hypothesized that exogenous PC may influence the evolution of inflammatory reaction in collagen-induced arthritis (CIA), a major murine model of RA [16,17]. Our primary aim was to explore the consequences of dietary PC supplementation on certain *in vivo *inflammatory parameters. To this end, we characterized the leukocyte–endothelial cell interactions and perfusion characteristics in the synovial microcirculation [18], and compared the effectiveness of oral PC pretreatment with that of PC therapy when the treatment protocol was initiated only after the occurrence of signs of inflammatory disease.

The study additionally extended to the effects of PC supplementation on endogenous cannabinoid receptor activation in the synovia. TNFα, endothelial nitric oxide synthase (eNOS) and inducible nitric oxide synthase (iNOS) expression levels were chosen as further endpoints to characterize the modulation of the anti-inflammatory potential of the nutrition protocols.

## Materials and methods

### Animal model

The experiments were performed on 50 male DBA1/J mice kept under specified pathogen-free conditions in isolated ventilated cages with a 12-hour light/dark cycle. The experimental protocol was approved by the local animal rights protection authorities and followed the National Institutes of Health guidelines for the care and use of laboratory animals.

At the age of 8 weeks, mice were immunized intradermally at the base of the tail with 50 μl bovine collagen II (2.5 mg/ml (Chondrex, Redmond, WA, USA) emulsified in 50 μl complete Freund's adjuvant (CFA) (4 mg/ml,; DIFCO, Detroit, MI, USA) in order to induce CIA (groups 1 to 3), or received CFA only (control groups 4 and 5). A second, booster immunization was performed 3 weeks later, when 50 μl incomplete Freund's adjuvant was administered with or without the same volume of collagen II. The animals were randomly allocated into the experimental groups. In group 1 (n = 10), the animals were immunized with collagen II in CFA, and were then kept on water and standard laboratory chow *ad libitum *(Ssniff Spezialdiäten GmbH, Soest, Germany). The mice were observed for 6 weeks after the first immunization. The animals in group 2 (PC_pre_, n = 10) received standard laboratory chow containing 1% PC (S-45, a phospholipid fraction isolated from soybean lecithin; Lipoid GmbH, Ludwigshafen, Germany) from the first immunization until the end of the experiments. In group 3 (PC_ther_, n = 10), the mice were kept on the normal diet until the onset of the inflammation (see below). At the appearance of the first signs of CIA, the animals were individually set onto the PC-enriched diet for 6 weeks. Group 4 (n = 10) and group 5 (n = 10) served as controls: these animals received either the normal diet (group 4) or the PC-enriched diet (group 5) without the induction of CIA. During the observation period, the food intake and the body weight of each animal were measured and registered daily.

### Clinical evaluation of collagen-induced arthritis

The scoring for CIA evaluation was performed in a blind manner by one investigator (GE) using the scoring system of Nandakumar and colleagues, which is based on the number of inflamed joints in each paw, inflammation being defined by swelling and redness [19]. Briefly, each inflamed toe or knuckle = 1 point, whereas an inflamed wrist or ankle = 5 points, resulting in a score from 0 to 15 (five toes + knuckles + one wrist/ankle) for each paw and from 0 to 60 points for each animal. Scoring was performed every second day during the observation period.

### Thermal stimulation

The animals were acclimatized to the experimental conditions for 1 hour preceding the test. They were then placed onto a heating plate set to 40°C to assess their thermal hypersensitivity. After 10 minutes, the positions of the limbs were rated three times on a numeric scale during a 15-minute period, according to the method initially described by Attal and colleagues [20] and used by our group [21]. The scores are as follows: 0 = the paw is pressed normally on the floor; 1 = the paw rests lightly on the floor and the toes are in a ventroflexed position; 2 = only the internal edge of the paw is pressed on the floor; 3 = only the heel is pressed on the floor and the hindpaw is in an inverted position; 4 = the whole paw is elevated; and 5 = the animal licks the paw.

### Surgical intervention

At the end of the observation period, the mice were prepared for *in vivo *fluorescence microscopy (IVM). The animals were anesthetized with ketamine (90 mg/kg body weight) and xylazine (6 mg/kg) and were placed on a heating pad to maintain a body temperature of 37°C. A catheter was inserted into the left jugular vein for fluorescent dye application. For IVM of the synovial microcirculation we applied the knee joint model, as described previously by Veihelmann and colleagues [22] and by our own group [16,17]. Briefly, the skin was incised and, after removal of the overlying soft tissues, the patella tendon was cut transversally and the proximal and distal parts were carefully mobilized. The Hoffa's fatty body was superfused with 37°C saline to prevent it from drying and was covered with a glass slide. The microcirculation was monitored after a 15-minute stabilization period.

### *In vivo *fluorescence microscopy

After intravenous injection of fluorescein isothiocyanate-labeled dextran (15 mg/kg; Sigma, Deisenhofen, Germany) and rhodamine 6 G (0.15 mg/kg; Sigma), IVM was performed with a Zeiss microscope (Axiotech vario 100 HD; Carl Zeiss, Jena, Germany) equipped with a 100 W mercury lamp and filter sets for blue (excitation/emission 465 to 495 nm/>505 nm) and green (510 to 560 nm/>575 nm) epi-illumination. Through the use of water-immersion objectives (×20 and ×40 W/numerical aperture 0.8; Zeiss), final magnifications of ×306 and ×630 were achieved. Images were recorded by means of a charge-coupled device video camera (FK 6990-IQ-S; Pieper, Schwerte, Germany) and transferred to an S-VHS video system for subsequent off-line analysis. At the end of the experiments, the animals were killed with an overdose of ketamine. Two limbs and lymph nodes were removed for further histological and molecular biological analysis.

### Microcirculatory analysis

For quantitative off-line analysis, a computer-assisted image analysis system was used (CapImage v7.4; Zeintl, Heidelberg, Germany). The functional capillary density was defined as the total length of red blood cell-perfused capillaries per observation area (cm/cm^2^). For assessment of the leukocyte–endothelial cell interaction in the postcapillary venules, the flow behavior of the leukocytes was analyzed as concerns free-floating, rolling and adherent leukocytes. Rolling leukocytes were defined as those cells moving along the vessel wall at a velocity less than 40% of that of the leukocytes at the centerline, and were expressed as a percentage of the total leukocyte flux. Venular leukocyte adherence was defined as the number of leukocytes not moving or detaching from the endothelial lining of the venular vessel wall during an observation period of 20 seconds. On the assumption of cylindrical microvessel geometry, leukocyte adherence was expressed as nonmoving cells per endothelial surface (n/mm^2^), calculated from the diameter and length of the vessel segment analyzed. The centerline red blood cell velocity in the postcapillary venules was determined by the line shift method.

### Histological assessment of arthritis

Limbs were placed *in toto *in 4% phosphate-buffered formaldehyde solution for 1 day, and then transferred to ethylenediamine tetraacetic acid solution for an additional 8 weeks to decalcify the bones. The tissue was next embedded in paraffin, sectioned (6 μm) and stained with H & E. Histological analysis was performed on coded sections by two independent investigators (GE and BV), using a semiquantitative histological score [23,24] as follows: inflammatory reactions in the synovial tissue (enlargement of the lining layer and the cellular density of the synovial stroma), 0 to 3 points; leukocyte infiltration of the joint, 0 to 3 points; inflammation-related cartilage damage, 0 to 3 points; subchondral bone erosion, 0 to 3 points.

### Real-time PCR analysis of cannabinoid receptors 1 and 2

The paws and lymph nodes of the mice were removed after IVM. Snap-frozen paws were homogenized with a mortar and pestle, and lymph nodes were homogenized with FastPrep instruments. Total RNA was extracted with the RNeasy Mini Kit (Qiagen, Hilden, Germany) according to the manufacturer's instructions. For reverse transcription, 300 U SUPERSCRIPT™ RNase H^- ^Reverse Transcriptase, 20 U RNasin, 3 μM random hexamers (Amersham Pharmacia Biotech, Uppsala, Sweden), deoxynucleoside triphosphate, dithiothreitol and 2 μg RNA sample per 25 μl reaction volume were used.

Gene quantification was performed on the ABI Prism 7700 Sequence Detection System (Perkin-Elmer Applied Biosystems, Weiterstadt, Germany). TaqMan primers and probes were purchased from Perkin-Elmer Applied Biosystems. Quantitative PCR was carried out with 50 ng cDNA according to the manufacturer's instructions in a final volume of 12.5 μl. The thermal cycling conditions were as follows: 2 minutes at 50°C, 10 minutes at 95°C followed by 45 to 50 repeats of 15 seconds at 95°C, and 1 minute at 60°C. In each run, a negative control (distilled water) was included. For each RNA isolation, measurements of gene expression were made twice, and the mean of these values was used for further analysis. According to the manufacturer's instructions (Applied Biosystems, Foster City, CA, USA), the comparative cycle threshold (C_t_) method and the internal control (GAPDH) were used to normalize the expression levels of target genes.

### PCR analysis of endothelial and inducible nitric oxide synthase and TNFα

Total RNA was extracted with the RNeasy Mini Kit (Qiagen) according to the manufacturer's instructions. RNA concentration was determined spectrophotometrically. First-strand cDNA was prepared by the reverse transcription of 1 μg total RNA, using the oligo (dT)_18 _primer (Biolabs, Frankfurt am Main, Germany) and Superscript II RNaseH-Reverse Transcriptase (Invitrogen, Karlsruhe, Germany) in the presence of dNTPs, 5 × first-strand buffer and dithiothreitol at 72°C for 10 minutes and 42°C for 60 minutes. The reverse transcriptase was inactivated at 95°C for 5 minutes.

Mouse eNOS was amplified by 35 cycles of PCR consisting of 94°C (30 s) for denaturation, 68°C (30 s) for primer-specific annealing and 72°C (30 s) for extension, using *Taq*DNA polymerase (Amersham Bioscience, Piscataway, NJ, USA). The following intron-spanning primers were used: 5'-AAG ACA AGG CAG GGG TGG AA-3' and 5'-GCA GGG GAC AGG AAA TAG TT-3'. Mouse iNOS was amplified by 30 cycles of PCR (described above). The following primer sequence was applied: 5'-ACC CCT GTG TTC CAC CAG GAG ATG TTG AA-3'; the reverse primer sequence was 5'-TGA AGC CAT GAC CCT TCG CAT TAG CAT GC-3'. For TNFα, the primers were 5'-GGC AGG TCT ACT TTG GAG TCA TTG C-3' and 5'-ACA TTC GAG GCT CCA GTG AAT TCG G-3'.

In a comparable assay, the RNA integrity and cDNA synthesis were tested using mouse GADPH as a housekeeping gene and the following primers: 5'-AAC GAC CCC TTC ATT GAC-3' and 5'-TCC ACG ACA TAC TCA GCA C-3'. In parallel, controls with H_2_O instead of DNA were carried out for every PCR reaction.

The PCR products were separated by electrophoresis on 2.0% agarose gels. Ethidium bromide-stained bands were visualized by UV illumination and were semiquantified densitometrically (TotalLab; Nonlinear Dynamics, Newcastle upon Tyne, UK). The expressions of these genes are referred to that of GADPH.

### Statistical analysis

Data analysis was performed with the SigmaStat for Windows statistical software package (Jandel Scientific, Erkrath, Germany). Nonparametric methods were used. Friedman repeated-measures analysis of variance on ranks was applied within the groups, followed by Dunn's method for time-dependent differences from the baseline. Differences between groups were analyzed with Kruskal–Wallis one-way analysis of variance on ranks, followed by Dunn's method for pairwise multiple comparison. In the figures, median values and the 25th and 75th percentiles are given. *P *< 0.05 was considered statistically significant.

## Results

### Food consumption and body weight changes

The food intake of the control groups remained constant, while the consumption decreased significantly in the groups immunized with collagen II (data not shown). The consumption of the PC-enriched chow significantly surpassed that of the normal food in the nontreated CIA mice and control animals (data not shown). The calculated PC consumption did not differ between the groups, ranging from 1.4 to 1.6 mg/day/g body weight.

### Incidence of collagen-induced arthritis

Clinical signs of arthritis were absent in the control groups. The incidence of CIA did not differ significantly between the treated groups, at 90% in group 1 (normal food), 80% in group 2 with the PC-enriched diet, and 90% in group 3 where the animals received the PC-enriched diet after the onset of inflammation.

### Severity of collagen-induced arthritis

**Figure 1:**
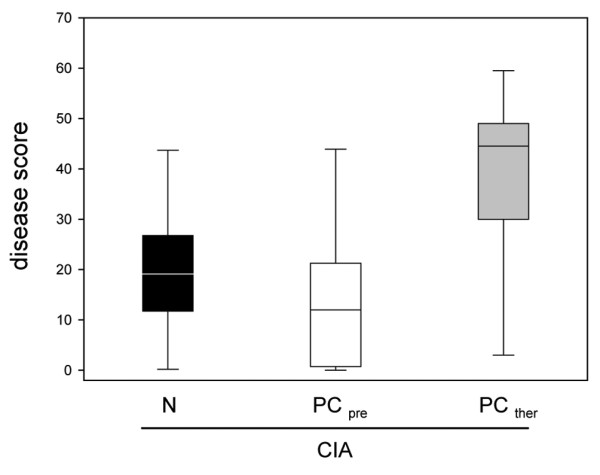
Clinical disease scores in animals with collagen-induced arthritis and diet supplementation. Clinical disease scores in animals with collagen-induced arthritis (CIA) and supplementation of either the normal diet (N) or the phosphatidylcholine-enriched diet, starting either with CIA induction (PC_pre_) or with the clinical onset of the disease (PC_ther_). For the induction of CIA, animals were immunized twice with collagen II and complete Freund's adjuvant/incomplete Freund's adjuvant. Three weeks after the second immunization, the clinical disease scores were determined as described in Materials and methods. Values given as medians with the 25th and 75th percentiles.

The clinical signs of inflammation appeared after the second immunization and there was a continuous progression until the end of the observation period. More limbs were affected in animals of group 1 than of group 2, suggesting moderate inflammation, but the difference between the groups was not significant (Figure 1). The animals in group 3 exhibited serious arthritis despite the PC therapy.

### Thermal hypersensitivity

**Figure 2:**
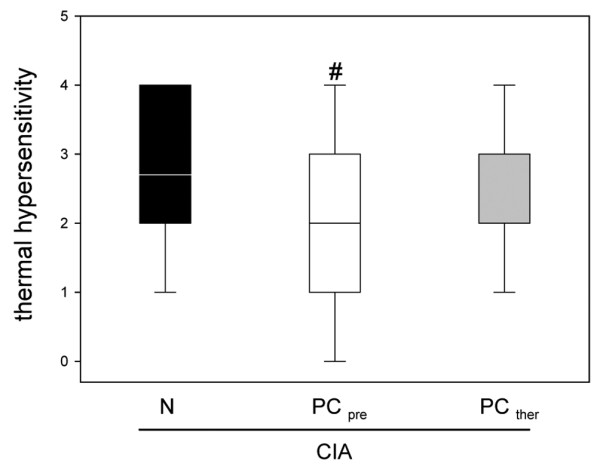
Thermal hypersensitivity of hind paws in animals with collagen-induced arthritis. Thermal hypersensitivity of hind paws in animals with collagen-induced arthritis (CIA) and either the normal diet (N) or the phosphatidylcholine-enriched diet, starting either with the CIA induction (PC_pre_) or with the clinical onset of the disease (PC_ther_). For the induction of CIA, animals were immunized twice with collagen II and complete Freund's adjuvant/incomplete Freund's adjuvant. Three weeks after the second immunization, the thermal hypersensitivity was assessed, as described in Materials and methods. Values given as medians with the 25th and 75th percentiles. #*P *< 0.05 versus N/CIA.

The inflammatory process associated with CIA was accompanied by secondary hyperalgesia (Figure 2). The dietary PC pretreatment in group 2 resulted in a statistically significantly lower thermosensitivity. This effect was not observed in group 3, where the PC-enriched diet was started after the onset of the inflammation (Figure 2).

### Microcirculatory changes in collagen-induced arthritis

**Figure 3:**
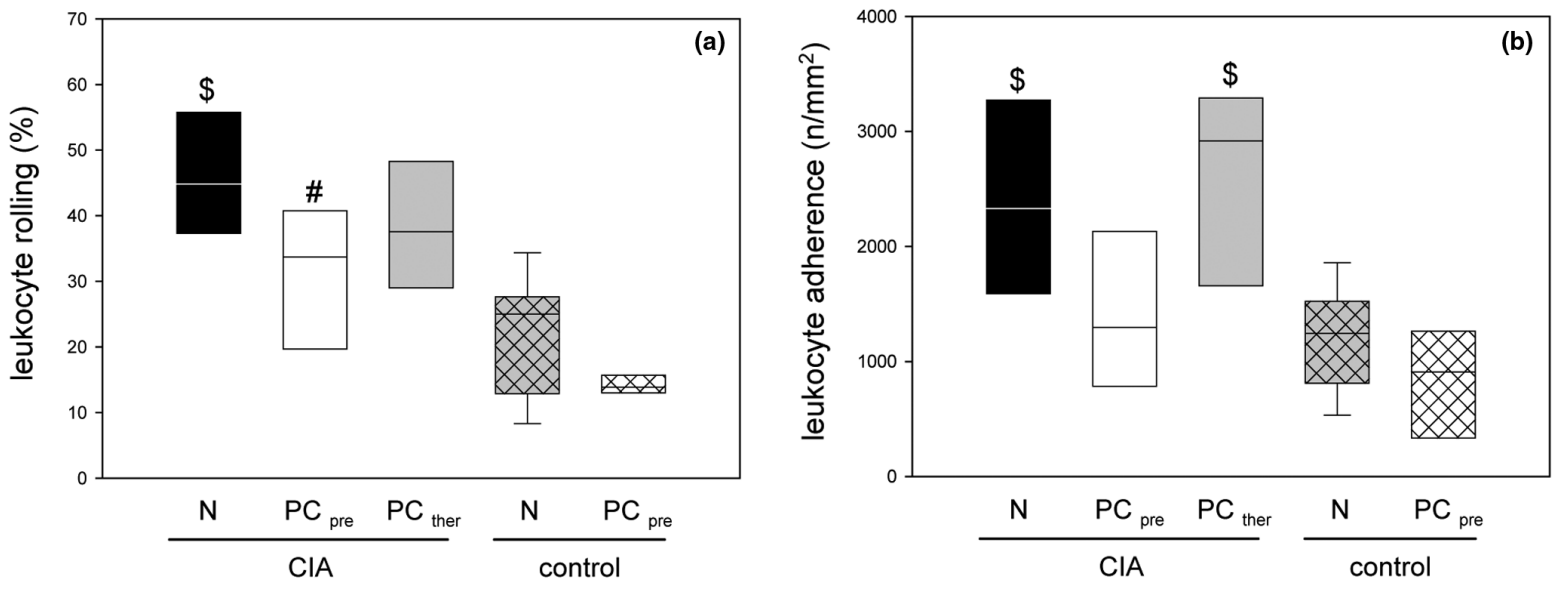
Primary and secondary leukocyte–endothelial cell interactions in synovial venules of animals with collagen-induced arthritis. Quantitative analysis of **(a) **primary (rolling) and **(b) **secondary (firm adherence) leukocyte–endothelial cell interactions in the synovial venules of animals with collagen-induced arthritis (CIA) and either the normal diet (N) or the phosphatidylcholine-enriched diet, starting either with the CIA induction (PC_pre_) or with the clinical onset of the disease (PC_ther_). For the induction of CIA, animals were immunized twice with collagen II and complete Freund's adjuvant/incomplete Freund's adjuvant. Three weeks after the second immunization, the knee joints were assessed by intravital fluorescence microscopy, as described in Materials and methods. Values given as medians with the 25th and 75th percentiles. #*P *< 0.05 versus N/CIA. $*P *< 0.05 versus N/control.

Local inflammatory injury was manifested in significant increases of both primary interaction (rolling) and secondary interaction (firm adherence) of PMN granulocytes with the microvascular endothelium. The microcirculatory analysis of the knee joints revealed a high percentage of rolling leukocytes in the postcapillary venules in group 1 (Figure 3a). The rolling fraction was significantly lower in the animals in group 2, which received the PC-enriched dietary pretreatment, but only slightly lower in the animals therapeutically treated. The loose interaction between the PMN granulocytes and the endothelium was less frequent in the control group (normal food) and was almost absent in control group 5 (PC-enriched food) (Figure 3a).

Concomitantly, the synovial venules of the knee joints in the CIA animals displayed a high number of firmly adherent leukocytes when compared with the control animals (≤ 1,000 cells/mm^2^) (Figure 3b). The early PC intake significantly decreased this reaction toward the control values, whereas the CIA animals with a late PC uptake did not benefit from the dietary supplementation, as revealed by the leukocyte adherence of approximately 3,000 cells/mm^2 ^(Figure 3b). The centerline red blood cell velocity in the synovial venules did not differ markedly in the CIA groups, ranging between 0.7 and 1.1 mm/s. The levels in the control groups were slightly lower (~0.5 mm/s).

**Figure 4:**
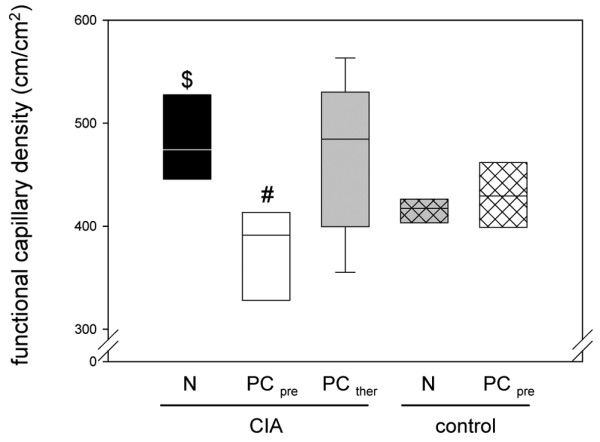
Functional capillary density in synovial tissue of animals with collagen-induced arthritis. Quantitative analysis of the functional capillary density in the synovial tissue of animals with collagen-induced arthritis (CIA) and either the normal diet (N) or the phosphatidylcholine-enriched diet, starting either with the CIA induction (PC_pre_) or with the clinical onset of the disease (PC_ther_). For the induction of CIA, animals were immunized twice with collagen II and complete Freund's adjuvant/incomplete Freund's adjuvant. Three weeks after the second immunization, the knee joints were assessed by intravital fluorescence microscopy, as described in Materials and methods. Values given as medians with the 25th and 75th percentiles. #*P *< 0.05 versus N/CIA. $*P *< 0.05 versus N/control.

Analysis of the functional capillary density revealed high values in the CIA animals in both group 1 and group 3 (Figure 4). In contrast, the early PC treatment prevented the CIA-associated neovascularization, as the functional capillary density in these animals (group 2) was comparable with or even somewhat lower than that in the controls (groups 4 and 5; Figure 4).

### Histological changes

**Figure 5:**
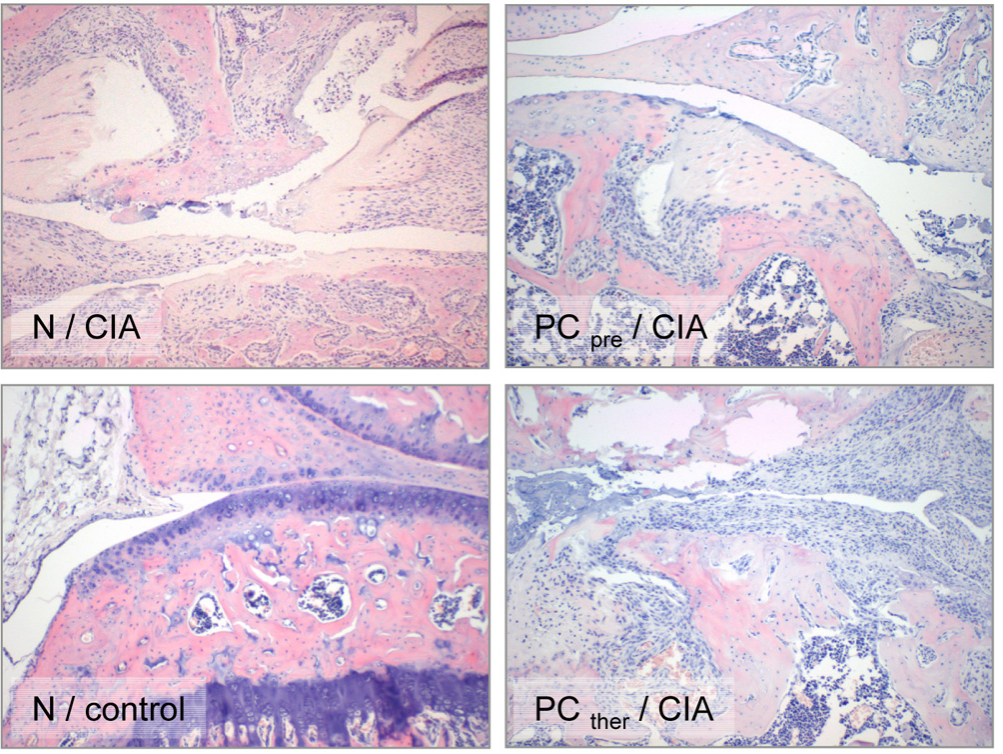
Photomicrographs with inflammatory reactions in knee joints of animals from groups 1 to 4. Photomicrographs of knee joints from groups 1 to 4 (H & E staining). N/CIA: group 1 (collagen-induced arthritis (CIA) + normal diet (N)) with synovitis, leukocyte infiltration and moderate cartilage erosions (original magnification ×10). PC_pre_/CIA: group 2 (CIA + pretreatment with the phosphatidylcholine-enriched diet) with moderate inflammatory reactions and slight damage to the cartilage (original magnification ×10). N/control: group 4 (control group with the normal diet), a knee joint with intact structure (original magnification ×10). PC_ther_/CIA: group 3 (CIA + the phosphatidylcholine-enriched diet started with the clinical onset of the disease) with serious inflammatory reactions, extensive cartilage destruction and subchondral bone damage (original magnification ×10). Group 5 (PC_pro_/control) is not presented since no relevant difference was found in the histological appearance of the two control groups.

**Figure 6:**
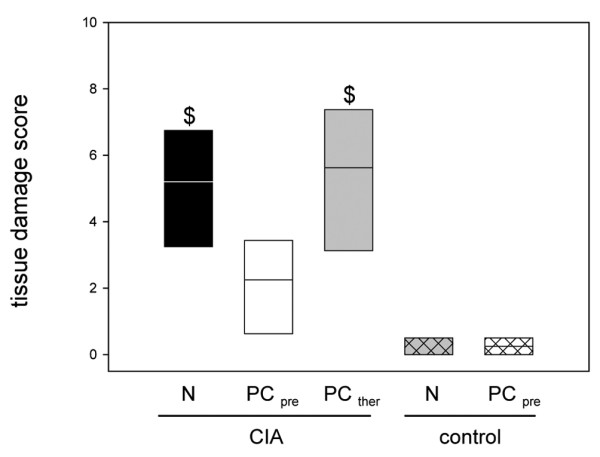
Histological changes in the knee joints of animals with collagen-induced arthritis. Semiquantitative scoring of the histological changes in the knee joints of the animals with collagen-induced arthritis (CIA) and either the normal diet (N) or the phosphatidylcholine-enriched diet, starting either with the CIA induction (PC_pre_) or with the clinical onset of the disease (PC_ther_). For the induction of CIA, animals were immunized twice with collagen II and complete Freund's adjuvant/incomplete Freund's adjuvant. Three weeks after the second immunization, the knee joints were removed, decalcified, sectioned and stained, as described in Materials and methods. Values given as medians with the 25th and 75th percentiles. $*P *< 0.05 versus N/control.

The light microscopic evaluation demonstrated the development of a serious inflammatory reaction in the CIA animals. Synovitis, cartilage and bone erosions were regularly detected in group 1, while the tissue damage in group 2 was less severe, with levels not significantly different from those for the control groups (Figures 5 and 6). Late PC therapy did not decrease the severity of the lesions (Figures 5 and 6).

### RNA expression of cannabinoid receptors 1 and 2, TNFα and inducible and endothelial nitric oxide synthase

The expressions of cannabinoid receptors 1 and 2 did not differ markedly between the groups and were not influenced appreciably by inflammation, PC pretreatment, or PC therapy (data not shown).

Both groups receiving the PC-enriched diet (either as prevention or as therapy) manifested a slight, but not significant decrease in the expression of TNFα as compared with group 1 (group 1: mean = 0.69, 25th percentile = 0.57, 75th percentile = 0.92; group 2: mean = 0.57, 25th percentile = 0.46, 75th percentile = 0.67; group 3: mean = 0.57, 25th percentile = 0.47, 75th percentile = 0.82). The TNFα expression in the control groups, however, did not differ significantly from those in the groups with inflammation (group 4: mean = 0.81, 25th percentile = 0.65, 75th percentile = 0.89; group 5: mean = 0.87, 25th percentile = 0.63, 75th percentile = 1.4).

**Figure 7:**
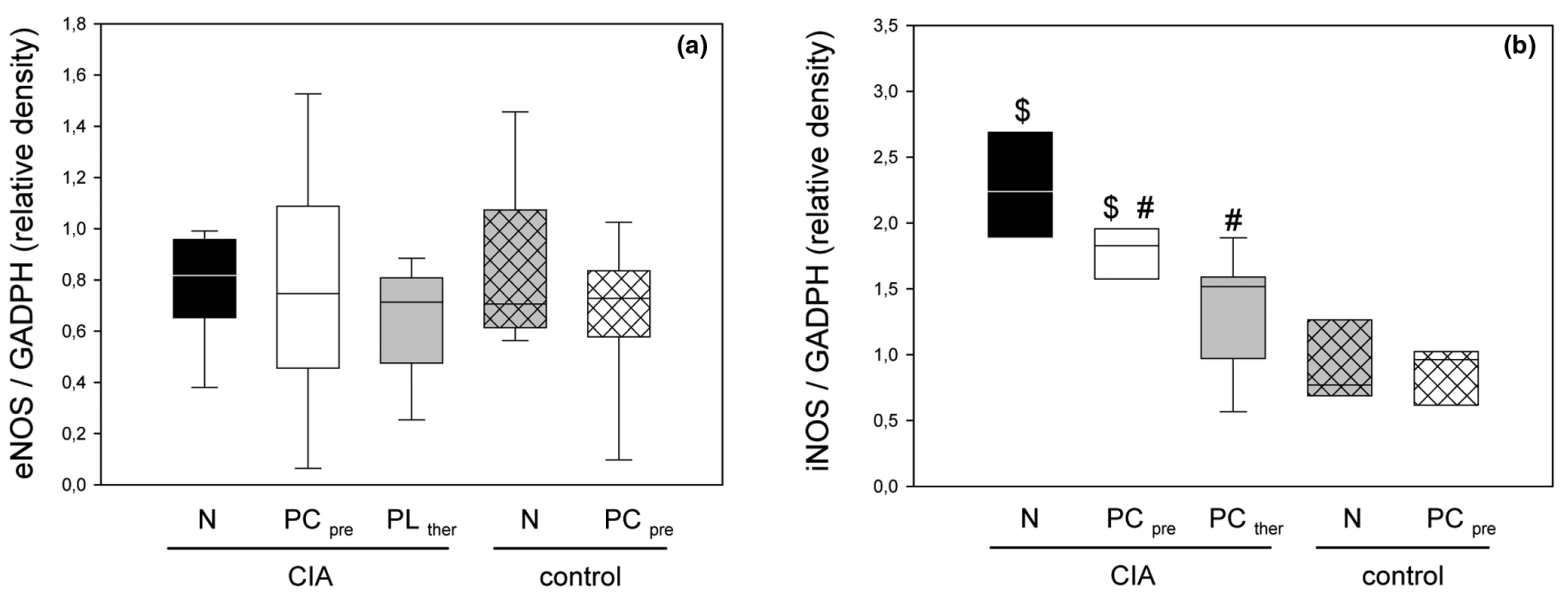
mRNA expression of endothelial and inducible nitric oxide synthase in animals with collagen-induced arthritis. mRNA expression of **(a) **endothelial nitric oxide synthase (eNOS) and **(b) **inducible nitric oxide synthase (iNOS) in the inguinal lymph nodes of animals with CIA and either the normal diet (N) or the phosphatidylcholine-enriched diet, starting either with the CIA induction (PC_pre_) or with the clinical onset of disease (PC_ther_). For the induction of CIA, animals were immunized twice with collagen II and complete Freund's adjuvant/incomplete Freund's adjuvant. Three weeks after the second immunization, lymph nodes were taken from the inguinal region and prepared as described in Materials and methods. The expressions of the target genes were referred to that of GADPH. Values given as medians with the 25th and 75th percentiles. #*P *< 0.05 versus N/CIA. $*P *< 0.05 versus N/control.

The level of eNOS expression in group 1 did not differ statistically significantly from those in the control groups, and the PC-enriched nourishment did not result in significant changes in eNOS expression (Figure 7a). In contrast, the expression of iNOS was considerably stimulated by the collagen-induced inflammatory challenge as compared with the control groups (Figure 7b). Both groups receiving the PC-enriched diet gave decreased values, and the attenuation of iNOS expression after PC therapy was somewhat more expressed as compared with that in the PC-pretreated group 2 (Figure 7b).

## Discussion

The prevalence of arthritis-attributed work limitation is very high and the number of affected people is rising steadily [25-28]. Efficient inflammatory control is of utmost importance, but therapy with traditional disease-modifying antirheumatic drugs is accompanied by a high incidence of side effects that hamper or even preclude the drugs' prolonged use. Prevention or early-stage treatment is therefore the primary goal [29], and in this respect the beneficial effect of nutritional components is of special interest. Indeed, it has already been shown that the oral intake of omega-6 and omega-3 fatty acids improves the symptoms of RA and diminishes the use of nonsteroidal anti-inflammatory drugs [30,31]. Dietary fatty acids bound to the glycerol backbone of phospholipids may be important sources of prostanoids, although it has been reported that water-soluble metabolites originating from the hydrophilic head-group of phospholipids also exert anti-inflammatory, protective properties [15].

The present results provide evidence that an increased dietary PC uptake prior to CIA is associated with significantly enhanced anti-inflammatory protection. CIA is a widely used, standardized tool for the investigation of chronic, autoimmune RA with polyarthritis and subsequent cartilage and bone erosions [32]. In our experiments, the RA model provided accurate measures for clinical and histological signs of joint inflammation and for simultaneous quantification of the microhemodynamics in the synovial microcirculation. The effects of PC intake were observed at different stages of the disease, and the results revealed that prophylactic oral PC supplementation ameliorated the CIA-induced pain and many of the clinical signs of inflammation. Moreover, histological evaluation indicated considerably improved arthritic conditions. On the other hand, serious inflammatory reactions developed after therapeutic PC administration – that is, when the dietary supplementation was started only after the onset of the disease. In this case, the signs of inflammation persisted, and hypersensitivity to pain and histological progression were also present. Taken together, these findings testify that orally administered PC is able to interfere with inflammation, but the critical time for PC involvement is during the onset of CIA.

The exact pathophysiology of RA remains uncertain, but the available evidence indicates that PMN migration and subsequent cytokine production induce synovial proliferation, with secondary cartilage and bone damage [33]. This conclusion has been reinforced further by the report that PMN infiltration contributes significantly to the developing joint inflammation during experimental RA [5]. In our study, microcirculatory examinations and histological evaluation were utilized to characterize the PMN-associated inflammatory reactions in the phase of RA evolution. Significantly enhanced leukocyte–endothelial interaction was detected in the synovial postcapillary venules, and the density of perfused microvessels was also increased. The present study did not establish an exact mechanism by which PC or its metabolites protect the synovial microcirculation, but it seems that the PC-enriched diet decreases the inflammatory activation of the PMN leukocytes. Indeed, PC is taken up by phagocytic cells [8], and accordingly it may accumulate in inflamed tissues, where the phagocyte function is altered by PC supplementation [34]. Other *in vitro *data have shown that dipalmitoylphosphatidylcholine modulates the inflammatory functions of monocytic cells [35] and that a mixture of PC and phosphatidylglycerol inhibits the respiratory burst and superoxide generation of human PMN granulocytes [36]. In the present study, we have found that firm leukocyte adhesion to the endothelial layer was effectively diminished by PC pretreatment. Overall, these results suggest a potential for early PC administration to decrease PMN activation.

The higher functional capillary density is indicative that new-vessel formation during CIA and PC pretreatment decreased the inflammation-induced increase in functional capillary density. Angiogenesis is an important component of the pathogenesis of RA, and it has been shown that several cytokines, chemokines (for example, TNFα, IL-8, and so forth) and the hypoxic environment of the arthritic synovium may all lead to angiogenesis in this condition [37]. Limitation of angiogenesis is therefore considered to be another, indirect sign of the anti-inflammatory effect of PC pretreatment.

We attempted to elucidate the role of the mediators that are presumed to be involved in the inflammatory reaction or that may contribute to its limitation. As the cannabinoid receptor system in the synovium has been shown to be a potentially important therapeutic target for the treatment of RA-associated pain and inflammation [38], we focused on the expression of cannabinoid receptors 1 and 2. We also took into account the possibility that a Ca^2+^-dependent *N*-acyltransferase may transfer fatty acids from the *sn*-1 position of PC or other glycerophospholipids to the amino group of phosphatidylethanolamine, with the formation of *N*-acylphosphatidylethanolamines. The hydrolysis of these latter by Ca^2+^-independent, constitutively active phospholipase D leads to the release of phosphatidic acid and *N*-acylethanolamines [39]. Polyunsaturated *N*-acyl-ethanolaminess, including the *N*-arachidonyl derivative anandamide, are endogenous cannabinoid receptor agonists with strong cytoprotective and anti-inflammatory properties [39,40]. The present results were negative in that the inflammatory challenge did not influence the expressions of the cannabinoid 1 and 2 receptors significantly. Similarly, the dietary PC intake did not exert any effect. It therefore seems that the protective effects of PC are not mediated via the cannabinoid receptor system. Despite their relative importance, however, the cannabinoid receptors are far from being the only way to control inflammatory reactions, and cannabinoids may use other pathways than cannabinoid receptors [41]. Further studies with cannabinoid receptor antagonists are required to confirm or to rule out the role of the cannabinoid system in this setup.

RA is mediated by a number of cytokines, including TNFα, and TNF-blocking agents play an important part in the therapy of the disease [29,42]. Our results indicated that a PC intake before or after the onset of the disease does not influence the inflammation-related TNFα expression appreciably. A further finding was that the TNFα expression in the control groups did not differ significantly from that in the animals exposed to the challenge of CIA. The explanation of these results may be the different temporal expression patterns of cytokines and chemokines in CIA. It has been demonstrated in bovine type II collagen-treated mice that the TNFα expression peaks between day 21 and day 28 of the inflammation, which is then followed by a considerable decline by day 42 [43]. It has also been reported that anti-TNFα therapy has little effect on CIA, while anti-TNFα antibodies reduced the severity of inflammation in another arthritis model [44]. The experimental setup required that the control groups receive the carrier without inflammation-evoking agent, but CFA alone is also able to result in arthritis that is accompanied by increased TNFα expression [45]. The relatively higher expression of TNFα in the control groups may therefore be explained by the application of CFA, which served as an emulsifier and enhancer for collagen type II. As regards severity and duration, adjuvant-induced arthritis is a less severe inflammatory condition than CIA. Hence, the elevation of TNFα expression in the control groups can be considered a sign of subclinical inflammation, manifested only at the biochemical level without functional and structural changes.

NF-κB is constitutively activated in RA [46], and PC inhibits the TNFα-induced proinflammatory response involving NF-κB activation *in vitro *[47]. Accordingly, it seems plausible that an increased PC input does not affect TNFα synthesis directly, but influences NF-κB-related events.

iNOS is one of the possible targets in arthritis [48]. iNOS in the synoviocytes, macrophages and PMN granulocytes in the joints is known to be upregulated during inflammation [49], and some data suggest that iNOS activation in the chondrocytes is a key event in the induction of adjuvant arthritis. [50]. iNOS-derived NO has been implicated in several aspects of the inflammatory cascade, including plasma exudation and cell migration. It has recently been recognized that both fatty acids and PC inhibit *in vitro *nitric oxide generation by iNOS [51]. In line with these data, we earlier reported that intravenous PC treatment inhibited the iNOS activity in a canine model of experimental esophagitis [13]. Our present results demonstrate that both PC pretreatment and PC therapy considerably decreased the expression of iNOS *in vivo*, the inhibitory effect being attained at the level of gene expression. Others have described that iNOS is involved in RA and negatively affects the bone homeostasis. A decrease in iNOS expression can therefore be regarded as a sign of improvement [52]. We can hence state that PC contributes to the amelioration of CIA via the inhibition of iNOS expression. Since nitric oxide plays a role in angiogenesis [53], such an effect of PC can also explain the decreased degree of synovial angiogenesis.

There is a growing scientific rationale for the use of dietary PC supplementation as adjunctive treatment in inflammatory disorders. PC increases the anti-inflammatory and analgesic activity of nonsteroidal anti-inflammatory drugs in acute and chronic models of arthritis [54], but the beneficial effect of PC as a carrier molecule is usually ascribed to alterations in the transport and bioavailability of active drug substances. We have demonstrated in the present study that PC *per se *can limit the inflammatory reaction of joints, without the addition of other pharmacological agents. Considerable research efforts are currently focused on the factors responsible for the increased susceptibility to RA [55]. We propose that a PC-enriched diet may be an effective means of prevention of RA when identified risk factors of RA are present.

## Conclusions

A prophylactic PC-enriched diet decreased the inflammation-related pain, angiogenesis and structural damage to the joints, and these effects were accompanied by the inhibition of iNOS expression and reduced leukocyte activation. Whereas a PC intake initiated after the onset of the disease also decreased the iNOS expression, the other signs and parameters of chronic inflammation were not influenced. The exact mode of action of PC requires further study, but the effectiveness of this pretreatment regimen points to a preventive anti-inflammatory approach for the amelioration of joint damage in this murine model of RA.

## Abbreviations

CFA: complete Freund's adjuvant; CIA: collagen-induced arthritis; eNOS: endothelial nitric oxide synthase; H & E: hematoxylin and eosin; IL: interleukin; iNOS: inducible nitric oxide synthase; IVM: intravital microscopy; NF: nuclear factor; PC: phosphatidylcholine; PCR: polymerase chain reaction; PMN: polymorphonuclear; RA: rheumatoid arthritis; TNF: tumor necrosis factor.

## Competing interests

The authors declare that they have no competing interests.

## Authors' contributions

GE performed the clinical evaluation of CIA, the thermal hypersensitivity test, the IVM and the microcirculatory analysis; he contributed to the histological assessment and drafted the manuscript. SI and NS performed the PCR. MB raised the potential beneficial role of PC in RA, and revised the manuscript. BV designed the study, contributed to the histological assessment and also critically revised the manuscript.
